# Laparoscopic training in porcine models: a tool for continuous professional skill enhancement for paediatric surgeons

**DOI:** 10.1007/s00383-026-06510-7

**Published:** 2026-07-08

**Authors:** Marco Pensabene, Maria Patti, Fabio Baldanza, Francesco Grasso, Luca Cicero, Maria Sergio, Maria Rita Di Pace

**Affiliations:** 1 Pediatric Surgery Unit, Department of Health Promotion, Mother and Child Care, Internal Medicine and Medical Specialties G. D’Alessandro, University of Palermo, 90127 Palermo, Italy; 2 Istituto Zooprofilattico Sperimentale Sicilia, IZS, 90127 Palermo, Italy

**Keywords:** Laparoscopic training, Laparoscopy, Laparoscopic pediatric surgery, Animal model, Pediatric surgeon

## Abstract

**Purpose:**

Laparoscopic training is an established component of minimally invasive surgery education, and involves different tools: virtual reality, training box and in vivo models. The use of porcine models is the most similar to human anatomy. Its role is well known during the residency program, but its specific impact on paediatric surgery consultants performance is poorly explored.

**Methods:**

Six consultant surgeons attempted a consecutive 4-h a week laparoscopic training on porcine models for a total of 12 weeks, focusing on refinement of fine dissection, intracorporeal suturing, and management of intraoperative complications. Participants underwent pre- and post-training (respectively Period A and B) assessment of surgical effectiveness by assessment of mean operative time, intraoperative and postoperative complications on three commonly performed procedures: inguinal hernia repair, varicocelectomy and laparoscopic orchiopexy.

**Results:**

210 procedures were analysed, 109 before and 101 after the training. We observed a significant overall reduction in operative time for all procedures (*p* = 0.007); more precisely, inguinal hernia repair and varicocelectomy showed a significant reduction in operative time (*p* = 0.028 and *p* = 0.04 respectively), while no significant reduction in operative time for laparoscopic orchidopexy was observed (*p* = 0.2668). Mean age at surgical intervention was significantly reduced in period B for herniorrhaphy.

**Conclusion:**

Laparoscopic training in porcine models could be considered an effective tool to improve skills of paediatric surgery consultants. Its use could allow the improvement of laparoscopic confidence and effectiveness also in surgeons with an already acquired learning curve, supporting the systematic and continuous integration of such programmes into the professional development.

## Introduction

The evolution of surgical education from traditional apprenticeship models, to contemporary systems incorporating advanced experiential and didactic teaching methods has been driven by a confluence of factors, including the imperative for enhanced patient safety, efficient operating room utilization, and individualized trainee learning needs [1]. This paradigm shift has led to the integration of simulation-based education, artificial intelligence-assisted training, and structured curricula to complement the historical Halstedian approach [2, 3]. However, the introduction of robotic surgical systems in paediatric surgery presents unique challenges, particularly concerning the limited availability of dedicated training programs and the specialized skills required for intricate paediatric reconstructions in constricted surgical fields [4]. Moreover, the access to robotic surgery is still challenging in paediatric surgery, due to its costs and the specialized equipment required [5]. Thus, laparoscopic surgery remains an important option for specific surgical procedures, and the subsequent specific training programs should be part of each surgeon’s training. Consequently, developing effective and accessible training methodologies for paediatric laparoscopic procedures remains crucial for surgical residents and fellows [6]. Training programs must therefore offer safe and realistic environments to develop laparoscopic skills.

The use of animal models is considered the gold standard for acquiring and refining complex surgical skills before their application in clinical settings, particularly in paediatric laparoscopic surgery where precision and advanced techniques are paramount [1, 7]. Specifically, porcine models offer a highly realistic physiological and anatomical approximation to human pediatric anatomy, facilitating the acquisition of essential psychomotor skills and tissue handling techniques in a controlled environment [8]. Nevertheless, animal models should be used as the end of a training program involving box trainers and virtual reality tools, if available [6].

Although the role of training programs in surgical education has been largely studied in trainees and residents, its role in experienced surgeons and consultants has been poorly explored [9]. This retrospective study aims to evaluate the impact of an intensive laparoscopic training course on porcine models involving six experienced paediatric surgeons of a single Institution Pediatric Surgery Unit, measuring its impact on the surgical performance.

## Methods

We focused our attention on the pre- and post-training results, by assessment of outcomes related to procedures that are already well established and considered mastered by the six surgeons themselves, namely laparoscopic varicocelectomy, orchiopexy and inguinal hernia repair. For all these procedures, indications for surgery were respectively: III grade varicocele or II grade symptomatic varicocele (testicular hypotrophy or scrotal pain/discomfort); unpalpable testis in patients > 12 months; presence of unilateral /bilateral inguinal swelling consistent with uncomplicated inguinal hernia.

All of these procedures mentioned above were performed at ourPediatric Surgery Unit between January 2020 and December 2025, by the same 6 surgeons involved. In the study we excluded the procedures performed during the course, in order to reduce some bias. These were already known and well acquired procedures for all surgeons involved, with a well established learning curve in mininvasive surgery [10–15][16,17]. All procedures were carried-out with the same instruments and tools; all surgeons performed the same techniques as described beyond. The analyzed procedures were divided into two period groups: “A” performed before and “B” after the course. We analyzed: operative time, rates of intraoperative and early postoperative complications (intraoperative bleeding, postoperative bleeding and recurrence) rates of conversion to open surgery .

For these procedures, all surgeons followed the same protocols: a Palomo varicocelectomy was performed in all varicocele cases using 5 mm operative and optical ports, with en bloc ligation of the internal spermatic veins in case of type 1 varicocele, and ligation of the deferential vein if a type 3 varicocele was previously diagnosed [11, 12]; a laparoscopic single-stage vessels sparing orchidopexy, using 3 mm operative ports and a 5 mm optical one in patients with unpalpable testis [18]; if vanishing testis or testicular agenesis were detected, those procedures were excluded from the study. An intraperitoneal “double N” ligation of internal inguinal ring with 3.0 non resorbable suture in all patients with inguinal hernia [19].

### The program

The training program was carried out between September 2022 and December 2022, and consisted of a weekly 4-hours session for 12 weeks, with one animal model assigned to every two surgeons. Each surgeon performed an equal number and type of procedures. Two categories of procedures—considered simple and complex—were carried out. The procedures performed during the training program were selected with the aim of improving effectiveness and efficiency in specific surgical skills such as intracorporeal knot tying, approaching major vascular structures, and managing complications (bleeding, injury to adjacent organs). Specifically, the following procedures were performed: ovariectomy, uterine tube excisions, cholecystectomy and detrusorotomy as minor procedures; Nissen fundoplication, intracorporeal intestinal anastomosis, nephrectomy as major procedures.

For the practical activities, 36 swine were used, each weighing up to 35 kg and sourced from conventional farms. Before performing the procedures, each subject underwent a premedication protocol consisting of Medetomidine 20 mcg/kg administered intramuscularly and anesthetic induction with Zolazepam 5 mg/kg + Tiletamine 5 mg/kg, also administered intramuscularly.

Subsequently, orotracheal intubation was performed using a size 7 endotracheal tube, with anesthetic maintenance provided by isoflurane in oxygen. Intraoperative analgesia was ensured through intravenous administration of tramadol 2 mg/kg.

At the end of the surgical procedure, as required by current regulations and in full compliance with animal welfare standards, each subject was euthanised by intravenous administration of Tanax^®^, following confirmation of deep anaesthesia, thus preventing any form of suffering.

Throughout each procedure, the animals’ physiological conditions were continuously monitored using sensors to measure heart rate, oxygenation, oxygen saturation, and respiratory rate. All animals adequately maintained body temperature and cardio-respiratory parameters, with no evidence of major intraoperative complications.

All participants were previously instructed to maintain appropriate and respectful behavior toward the animal, in order to avoid any imprudent or negligent actions and to ensure the highest level of technical proficiency during the procedure.

The training program was previously authorized by the Ethics Committee – Animal Welfare Body of the IZS Sicilia, which issued a Positive Motivated Opinion. The documentation was subsequently submitted to the Italian Ministry of Health, which, after thorough evaluation, granted official authorization no. 674/2023-PR (Response to protocol 28875.41).

The protocol was designed in order to refine specific laparoscopic skills such as fine dissection, vessel management and intracorporeal knot tying. Namely, all procedures performed aimed to improve fine dissection, while cholecystectomy and nephrectomy aimed to improve mainly the approach to vascular structures; Nissen fundoplication and intestinal anastomosis aimed to improve intracorporeal knot tying.

For statistical analysis a Student’s T Test was used for comparison of ages at surgical intervention and operative time, while Fisher exact test was used for comparison of complications occurrence. Significance was assumed for *p* < 0.05 (C.I. 95%).

## Results

A total of 268 procedures were performed in the studied period. 25 of them, performed in the same period of the course, were excluded; moreover 19 laparoscopic explorations for unpalpable testis revealed vanishing or testicular agenesis, and thus were excluded. In order to obtain a more homogenous group of procedures, only monolateral herniorrhaphy were included, and thus, 14 patients with bilateral hernia were excluded. Therefore 210 procedures were included, of whom 109 performed before the training (group A) and 101 after the training (group B). More precisely, we compared a total of 91 procedures for inguinal hernia (45 group A and 46 in group B), 73 laparoscopic varicocelectomy (39 group A and 34 group B) and 46 laparoscopic orchidopexy (25 group A and 21 group B). Results are described in Table 1.

Mean age at surgical intervention was: 13,4 years (range 11,5–16,2) in patients undergoing varicocelectomy (13.9 group A, 13.6 group B); 57.3 months (range 24–156) for herniorrhaphy (64,3 group A, 50,2 group B); 15 months (range 11–17) for orchiopexy (14.6 group A, 13.7 group B).

Mean body weight at surgical intervention was 48 kg (range 32–78) for patients undergoing varicocelectomy, (46.5 kg period A, 50 period B), 23.6 kg (range 12–60 kg) for patients undergoing laparoscopic herniorraphy (25.9 kg period A, to 21.2 kg period B); 13 kg for patients undergoing laparoscopic orchiopexy (13.3 kg period A, 12.8 kg period B).

We observed no statistically significant differences between mean age and mean body weight at surgical intervention for varicocelectomy and orchiopexy, while a significant difference was found comparing mean age and mean body weight at surgical intervention for herniorrhaphy (respectively *p* = 0.0157 and *p* = 0.0438).

No major bleeding occurred in the analysed period in both groups. In 7 patients a minor, self-limiting bleeding was observed, respectively 5 in group A and 2 in group B, with no significant differences (*p* = 0.448); all of that minor bleeding occurred in varicocele surgery.

**Fig. 1:**
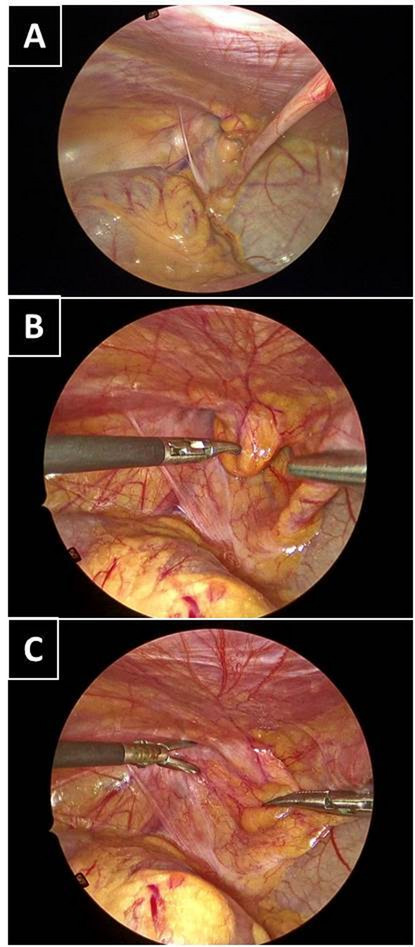
Laparoscopic view of a large lipoma protruding into the inguinal canal, nearly occluding the internal inguinal ring and impairing visualization of the anatomical landmarks, thus precluding the safe completion of laparoscopic inguinal hernia repair. **A** laparoscopic view, **B** and **C** the lipoma deeply protrude within the inguinal canal

**Fig. 2:**
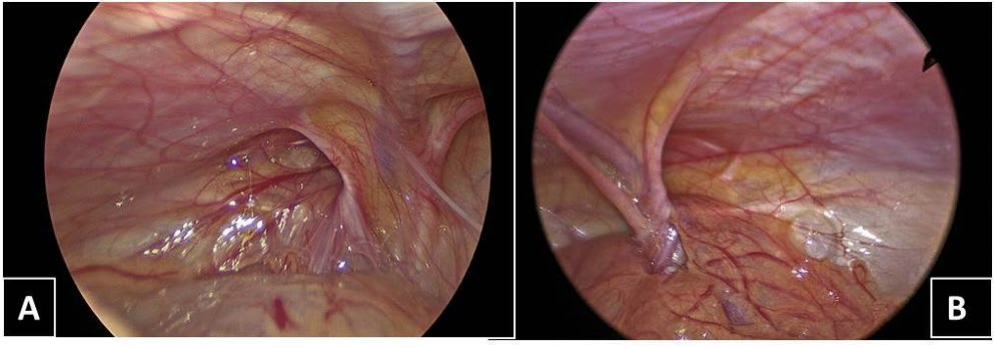
Laparoscopic view of markedly enlarged internal inguinal rings (> 2.5 cm), left (**A**) and right (**B**), respectively

In 4 males patients undergoing laparoscopic herniorrhaphy, conversion to open surgery was required, 3 in group A and 1 in group B (*p* = 0.361); in two of these patients, conversion to open surgery was required because of laparoscopic exploration revealed a huge lipoma that did not allow a clear visualization of the internal inguinal ring (Fig. 1). In the remaining 2 patients, of whom one patient had intraoperative observation of bilateral hernia, the mainly involved internal inguinal ring was too wide (> 2.5 cm) to ensure a tension-free closure of the internal inguinal ring [16][20] (Fig. 2).

Three patients experienced recurrent hernia, respectively 1 treated in the period A and two in the period 2.

No significant difference between the period A and B was observed in complication occurrence in laparoscopic inguinal hernia repair. No recurrent varicocele was observed in the analyzed period.

Analyzing intraoperative time we observed a significant reduction when comparing period A period B, respectively 59 min vs. 50 min (*p* = 0.007). More precisely, mean operative time for laparoscopic varicocelectomies reduced from 61 min to 49 min (*p* = 0.04), while laparoscopic hernia repair reduced from 58 min to 50 min (*p* = 0.028). Although a reduction in operative times was observed when comparing pre and post-course operative times of laparoscopic orchiopexy, respectively 91 min and 85 min, a significant difference was not observed (*p* = 0.2668). In six cases a two stage orchiopexy was performed, respectively 5 in the pre-course period, while 1 in the second period. Results are resumed in Table 2.

**Table 1:** Distribution of procedures among groups

	Period A	Period B	Total
Palomo	39	34	73
Herniorrhaphy	45	46	91
Orchiopexy	25	21	46
Total	109	101	210

**Table 2:** The table resumes the statistical findings of differences among groups in terms of mean age at surgical intervention, mean operative time and complication occurrence/outcome

		Global (range)	Period A	Period B	p value
Palomo	Mean age at surgical intervention (years)	13.4 (11.5–16.2)	13.9	13.6	0.541
	Mean body weight (kg)	48 kg ( 32–78)	46.5	50 kg	0.323
	Mean operative time (min)	55 (38–85)	61	49	**0.04**
	Complications/outcome comparison	7 (minor bleeding)	5 (minor bleeding)	2 (minor bleeding)	0.438
Herniorrhaphy	Mean age at surgical intervention (months)	57.3 (24–156)	64.3	50.2	**0.0157**
	Mean operative time (min)	54 (32–110)	58	50	**0.028**
	Mean body weight (kg)	23.6 (12–60)	25.9 (15–60)	21.2 (12–59)	**0.0438**
	Complications/outcome comparison	7 (4 conversion to open surgery, 3 recurrence)	4 (3 conversion to open surgery, 1 recurrence)	3 (2 recurrence, 1 conversion to open surgery)	0.714
Orchiopexy	Mean age at surgical intervention (months)	15 (11–17)	14.6	13.7	0.067
	Mean operative time (min)	87 (55–185)	91	85	0.2668
	Mean body weight (kg)	13 (11.5–17)	13.3	12.8	0.376
	Complications/outcome comparison	6 (two stage orchiopexy)	5 (two stage orchiopexy)	1 (two stage orchiopexy)	0.198

## Discussion

Education models involving the use of emerging technology and simulation are well established tools for refinement of surgical skills. It has been demonstrated that simulation-based approaches require the identification and definition of appropriate parameters in order to measure improvements in performance [21, 22]. Among the available simulation modalities, animal models—and porcine models in particular—remain the closest approximation to human anatomy. These models offer the realistic opportunity to manipulate tissues, address complications, and manage the physiological responses to surgical maneuvers in a real-world setting that is, at the same time, controlled.

While the value of porcine models in residency training is well established, their role in maintaining and enhancing the skills of experienced surgeons has been less thoroughly explored.

The present study specifically aims to focus on paediatric surgery consultants performing procedures already considered mastered that, although routinary performed, require refined skills such as precise dissection, secure intracorporeal knot tying, and careful handling of vascular structures.

We observed a reduction in operative times after completion of the training program in the overall cohort and, more specifically, in laparoscopic varicocelectomy and inguinal hernia repair.

Interestingly, laparoscopic orchidopexy did not show a significant reduction in operative time, despite a trend toward improvement. Initially one could interpret this observation as a consequence of the intrinsic complexity and variability of this procedure, particularly in cases of high intra-abdominal testis, where anatomical factors and testicular mobility play a major role. At the same time, we observed that two-stage procedures were more frequently performed in the pre-training group. On the contrary, in group B, two-stage procedures were rarely performed, with longer mean operative times, despite differences that were not significant. We speculated that the improved technical skills and confidence, allowed surgeons to prolong the laparoscopic dissection of intra-addominal testes, resulting in an effective one-time procedure, with non-significant differences in operative time.

Notably, the rate of intraoperative and postoperative complications did not differ significantly between the pre- and post-training periods. While this may initially appear counterintuitive, it is essential to interpret this finding in the context of the surgeons’ baseline expertise, and reflects the pre-existing learning-curve. In such a setting and a small sample of procedures and surgeons, expecting a significant reduction in complications rates may be unrealistic. However, with regard to laparoscopic orchiopexy, the absence of an increase in complications in group B, combined with an increased rate of one-time surgery, could be considered an improvement in surgical effectiveness.

From a merely speculative point of view, the significant reduction in patient’s age at surgery observed in herniorrhaphy, may be related to an enhanced surgeon confidence in handling reduced laparoscopic working spaces.

The reduction in minor bleeding events and conversions to open surgery in the post-training group, although not significant, further supports the hypothesis. Nevertheless, these trends should be confirmed in larger cohorts to obtain a more robust scientific significance.

These results suggest that, once a laparoscopic and endoscopic familiarity has been obtained, the improvement observed in the presented pool of experienced surgeons could be due to an increased surgical efficiency, likely reflecting enhanced dexterity, economy of motion, and decision-making speed rather than mere procedural familiarity.

This consideration reinforces the concept that surgical effectiveness is a dynamic process, since continuous exposure to complex laparoscopic tasks in a high-fidelity environment appears to facilitate skill refinement and consolidation, even after formal training has been completed. This finding is consistent with the concept of lifelong learning in surgery and challenges the traditional assumption that once competence is achieved, additional technical training yields limited benefits [22–24].[25,26]

In experienced surgeons, the integration of high-fidelity animal models allow integration of technical, cognitive, and non-technical skills in a realistic operative setting may be particularly valuable, as it enables reassessment of ingrained habits, correction of suboptimal techniques, and reinforcement of best practices [27–29].

In the presented paper we believe that the improvement in operative effectiveness observed could be related to an increase in readiness and confidence with the laparoscopic scenario, rather than a simple technical refinement. To this point, we support the concept that porcine models should represent the final step of a graduated training pathway, following box trainers and virtual reality simulators.

We acknowledge that these findings require confirmation in larger studies; however, their potential implications should be taken into consideration. Beyond individual improvements in surgical skill, integrating structured laparoscopic training into continuing professional development could yield benefits at the system level, such as more standardized quality of care, reduced variability between operators, and enhanced operating room efficiency. In paediatric surgery, where case volumes may be limited and procedural exposure uneven, with wide clinical variability, periodic high-intensity training sessions could compensate for variability in clinical practice and help maintain high standards of individualized care [30, 31].

In a scenario where robotic platforms are increasingly adopted but its limits remain (costs, availability of pediatric settings), laparoscopy is still a valuable approach in minimally invasive surgery. Porcine model–based training could represent a realistic and effective strategy to maintain advanced laparoscopic proficiency, particularly where access to robotic simulators is constrained.

Ethical considerations related to animal use remain an important topic. In this study, strict adherence to animal welfare regulations, ethical approval, and appropriate anesthetic and monitoring protocols ensured compliance with current standards. Nevertheless, the ethical justification for animal models should be continually reassessed, balancing educational benefits against ethical responsibility. In this context, limiting animal-based training to advanced stages of skill refinement and integrating it within comprehensive curricula may represent a responsible and effective approach.

Several limitations of the present study must be acknowledged.

Its retrospective design inherently limits causal inference and implies a lack of objective skill assessment. Moreover, time-related changes falsely appear to be an effect of the intervention, further limiting the study.

The relatively small sample size and the absence of a control group may have reduced the ability to detect differences in less frequent outcomes such as complications. Additionally, operative time, while a widely used surrogate marker of surgical efficiency, does not fully capture qualitative aspects of surgical performance.

However, the homogeneity of the surgical team and the standardized operative techniques employed strengthen internal validity and reduce confounding factors related to procedural variability.

The wide time-window analyzed could represent a potential limit, since surgical performance is expected to increase over time. Nevertheless, one should interpret this finding in the context of the surgeonspre-existing learning-curve. We assumed that, given the previous learning-curve of involved surgeons and the little variability, substantial benefits might become more evident expanding the number of procedures analyzed.

Furthermore, the study was conducted in a single institution, potentially limiting generalizability.

Multicentric prospective studies would be valuable to confirm our findings and define optimal training frequency, duration, and content for experienced surgeons; future studies should incorporate validated objective assessment tools, such as motion analysis or global rating scales, to provide a more comprehensive evaluation of skill acquisition.

In conclusion, this study suggests that intensive laparoscopic training on porcine models could be helpful in enhancing surgical efficiency even in experienced paediatric surgeons. The simulation-based training thus should not be reserved to novices, but should be part of the continuous professional development. In other words, the study aims to provide scientific support to the well-established principle that “*practice makes perfect*,” highlighting the concept that learning is a continuous process.

A structured and intensive laparoscopic training program based on porcine models could support surgeons to further refine and improve their performance and should be considered a component of the educational pathway of residents, early-career surgeons, and consultants.

## Data Availability

No datasets were generated or analysed during the current study.
